# Reorientational dynamics of amino acids in aqueous solution probed by ultrafast optical Kerr effect spectroscopy

**DOI:** 10.1080/14686996.2026.2736324

**Published:** 2026-09-21

**Authors:** Koichi Murakami, Tomohiro Nobeyama, Yuta Koda, Takahiro Sasamori, Kentaro Shiraki, Yohei Yamamoto, Soh Kushida

**Affiliations:** a Institute of Pure and Applied Sciences, University of Tsukuba, Tsukuba, Japan; b The Hakubi Center for Advanced Research, Kyoto University, Kyoto, Japan; c Tsukuba Research Center for Energy Materials Science (TREMS) and Hydrogen Boride Research Center, Tsukuba Institute for Advanced Research, University of Tsukuba, Tsukuba, Japan

**Keywords:** Amino acids, optical Kerr effect spectroscopy, reorientational dynamics

## Abstract

Reorientational relaxation of a solute in aqueous solution reflects the friction from its surroundings, yet observing solute-specific rotational motion at the microscopic level is difficult. Ultrafast optical Kerr effect (OKE) spectroscopy can follow molecular reorientational relaxation in liquid phase, but it is not obvious whether the slow picosecond component arises from the rotation of the solute itself or from water slowed by the increased viscosity when it is applied to aqueous solution systems. Here, we use OKE spectroscopy to investigate the reorientational dynamics of three amino acids (alanine, proline, and lysine monohydrochloride) in aqueous solution in terms of concentration, temperature, and chirality. Using poly(vinyl alcohol) (PVA) as a control, we found that increasing the macroscopic viscosity by approximately eightfold did not alter the reorientational relaxation time of water. This result indicates that OKE provides information on the local rotational friction associated with the solute and highlights its unique role as a technique complementary to rheometry, which measures macroscopic viscosity. This study provides new insight into the local rotational environment experienced by solutes in aqueous amino acid solutions.

## IMPACT STATEMENT

Non-resonant OKE, together with the viscosity controls, reveals amino acid associated reorientational dynamics and local rotational friction in aqueous solution beyond macroscopic viscosity.

## GRAPHICAL ABSTRACT

**Figure:**
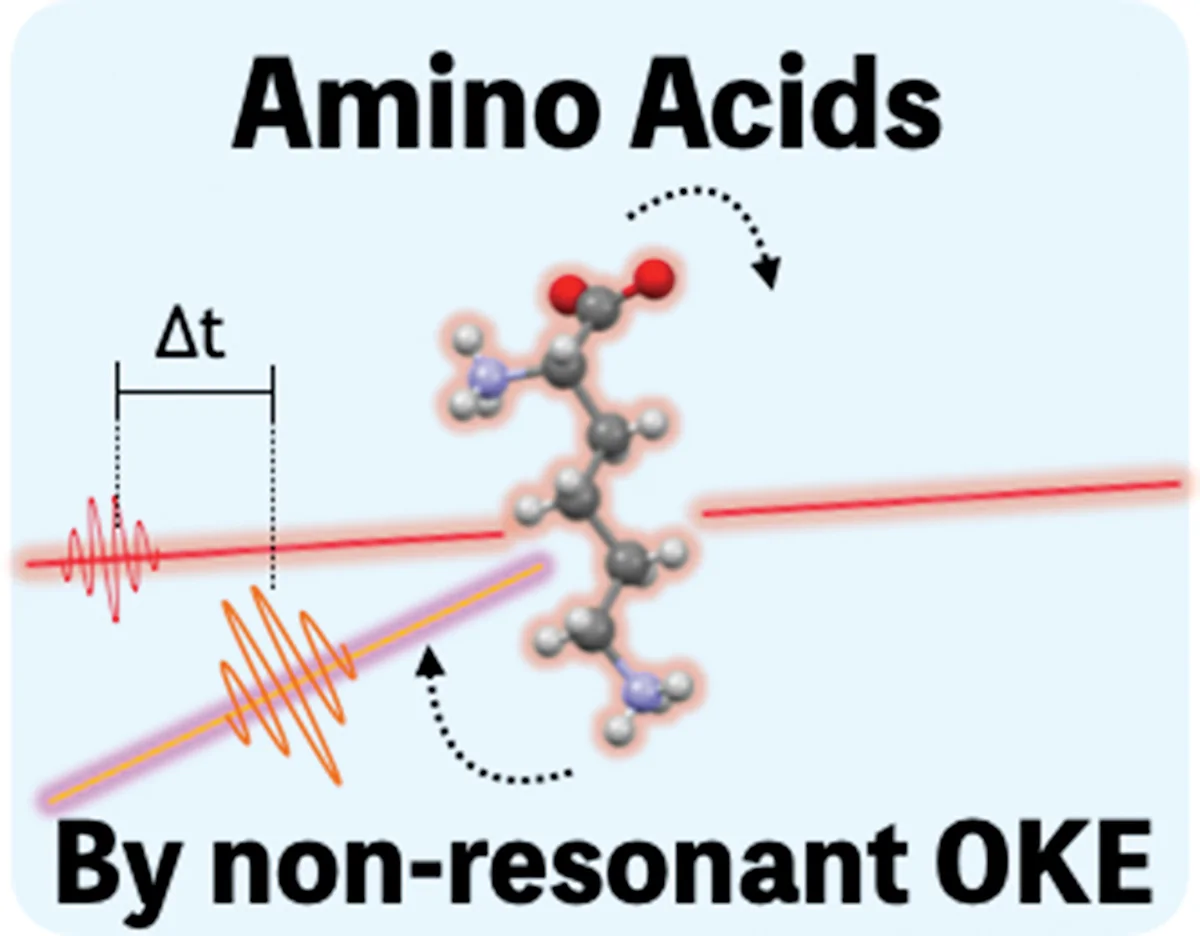

## Introduction

Molecules in solution continually change their orientation through thermal motion. The rate of this reorientation reflects the friction between solutes and their surroundings at the local environment. This has long been discussed in the framework of Stokes–Einstein–Debye relation [1]. Observing the microscopic rotational dynamics of an individual solute in aqueous solution is, however, difficult. Ultrafast optical Kerr effect (OKE) spectroscopy is a time-resolved nonlinear optical method that measures the transient birefringence induced by an ultrashort pump pulse; the decay of the OKE signal reflects the reorientational relaxation of liquid-phase molecules on the femto- to picosecond timescale [2]. OKE spectroscopy has been applied to a wide range of liquid systems, including simple liquids, ionic liquids, and liquid crystals [1,3]. However, most conventional OKE studies have focused on neat liquids. Direct investigations of solute reorientational dynamics remain comparatively limited because the non-resonant OKE response contains contributions from all components, making it difficult to distinguish the solute response from the generally more abundant solvent background. When solute-specific dynamics have been targeted, resonant OKE has therefore often been employed, in which the excitation wavelength is tuned to an electronic transition of the solute to enhance its contribution [4]. Although this resonant enhancement provides much greater sensitivity to the solute dynamics, the range of application is generally restricted to chromophoric molecules, particularly molecular dyes possessing a distinct absorption band. Consequently, resonant OKE is not readily applicable to nonchromophoric solutes, such as many small aliphatic molecules and amino acids, that lack a suitable electronic absorption band in the visible region. By contrast, only a limited number of studies have employed non-resonant OKE to investigate the reorientational dynamics of solutes in aqueous solution [1–3,5–14].

Most aqueous OKE studies have rather focused on how dissolved species perturb the structure and dynamics of the surrounding water. For example, Meech and co-workers have used OKE spectroscopy to elucidate how solutes such as urea, ionenes, and acetate modify the hydrogen-bond structure and dynamics of water [6,7,14]. Righini and co-workers investigated the temperature dependence of the reorientational dynamics of aqueous urea and showed that its relaxation behavior was consistent with the Debye–Stokes–Einstein framework [7]. Nevertheless, in a non-resonant OKE measurement of an aqueous solution, it is not immediately evident whether a slow picosecond component originates from the reorientation of the solute or from solvent water whose dynamics have been modified by the presence of the solute (Figure 1).

**Figure 1.**
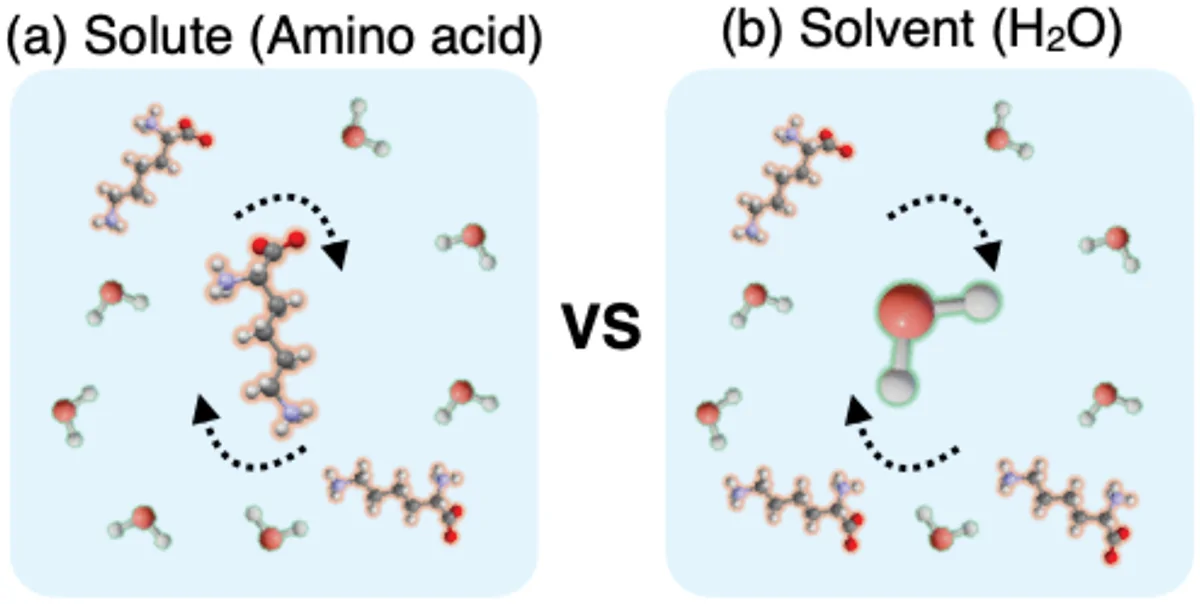
Two possible origins of the picosecond component in the OKE signal of an aqueous amino acid solution: reorientation of (a) the solute (amino acid) itself, or (b) the solvent water (slowed by the increased viscosity).

Although the OKE response of neat water is known to be weak and to decay within the sub-picosecond regime [8], it has not been established whether a slower picosecond component observed in aqueous solutions can be assigned to solute reorientation. For example, previous OKE studies of aqueous solutions have often assigned slow picosecond components to hydration-water or hydrogen-bond-network dynamics, reflecting the fact that non-resonant OKE detects the collective polarizability response of all components in solution [14]. At the same time, in concentrated solutions of optically anisotropic solutes, a solute-associated contribution can also become significant because the orientational OKE response depends strongly on molecular polarizability anisotropy. Therefore, whether a slow picosecond component mainly reflects hydration-water dynamics, solute-associated reorientational relaxation, or a coupled solute–solvent response must be evaluated for each system.

In this work, heterodyne-detected OKE spectroscopy is employed to investigate the reorientational dynamics of three amino acids (alanine, proline, and lysine monohydrochloride) in aqueous solution and to examine whether non-resonant OKE can resolve solute reorientation. Systematic measurements as functions of concentration, temperature, and chirality, together with control experiments using aqueous poly(vinyl alcohol) (PVA) solutions [3,6], support the assignment of the slow picosecond response to amino acid reorientation. These measurements establish the origin of the slow OKE response and provide insight into the local environment governing the rotational motion of amino acids in aqueous solution.

## Experimental methods

### Solubility measurements of amino acids

All the chemical compounds were purchased from either Merck, TCI, or Wako and used as received. The purities were as follows: L-alanine (>99.0%), D-alanine (>98.0%), L-valine (>98.0%), D-valine (single spot by TLC), L-arginine (>99.0%), D-arginine (single spot by TLC), L-phenylalanine (>98.0%), D-phenylalanine (>98.0%), L-proline (>98.0%), D-proline (>98.0%), L-lysine monohydrochloride (>98.0%), and D-lysine monohydrochloride (>98.0%). Solubility was determined from UV–Vis absorbance measurements using calibration curves constructed according to the Lambert–Beer law. Absorbance measurements were performed using a UV–Vis spectrophotometer (V-570, JASCO Corporation, Japan). Calibration curves were obtained by linear regression of absorbance versus concentration at the following wavelengths: alanine (207 nm), valine (207 nm), arginine (221 nm), phenylalanine (257 nm), proline (213 nm), and lysine monohydrochloride (217 nm). The concentrations used for calibration were 10, 20, and 50 mM for alanine, arginine, phenylalanine, proline, and lysine monohydrochloride, and 3, 6, and 15 mM for valine. Saturated aqueous solutions were prepared by adding an excess amount of each amino acid to water at room temperature and stirring for several hours to reach equilibrium. After equilibration, the suspensions were centrifuged at 10,000 g for 5 min to remove undissolved solids, and the supernatants were collected. The pH values of the supernatants were as follows: alanine (6.08), arginine (11.4), phenylalanine (5.73), lysine hydrochloride (5.48), proline (7.03), and valine (6.34). The supernatants were diluted 10-fold prior to measurement, except for racemic lysine monohydrochloride, which was measured without dilution. The solubilities were calculated using the corresponding calibration curve, taking into account the dilution factor.

### Optical Kerr effect spectroscopy

A schematic of the experimental setup is shown in Figure 2. Ultrafast OKE spectroscopy measurements were performed using a regenerative amplified Ti:sapphire laser system (Mai Tai and Solstice, Spectra-Physics, USA), delivering pulses with a duration of approximately 70 fs at a repetition rate of 1 kHz and a central wavelength of 800 nm. The output beam was split into pump and probe pulses. The pump pulses were generated by directing a portion of the fundamental output into an optical parametric amplifier (TOPAS, Light Conversion, Lithuania), producing tunable pulses centered at 694 nm. The pump beam polarization was adjusted to 45° with respect to the probe polarization using a polarizer. The pump beam was weakly focused onto the sample with a lens, yielding a typical beam diameter of approximately 300 μm at the sample position. The crossing angle between the pump and probe beams at the sample was kept below 10° to ensure effective spatial overlap. The probe beam was derived directly from the 800 nm fundamental output. It was focused onto the sample to a beam diameter of approximately 120 μm. Before the sample, a motorized polarizer (P1) and a precisely controlled quarter-wave plate (QWP) were installed. The transmission axis of P1 and the fast axis of the QWP were initially aligned at 0°. Heterodyne detection was implemented by rotating P1 by ±2° relative to this position and taking the differential signal between the two measurements [1,3,4,10]. This procedure enables sensitive detection of pump-induced birefringence with linear sensitivity to the third-order nonlinear polarization. Details of the heterodyne detection scheme and calibration procedures are described in our previous publication [4].

**Figure 2.**
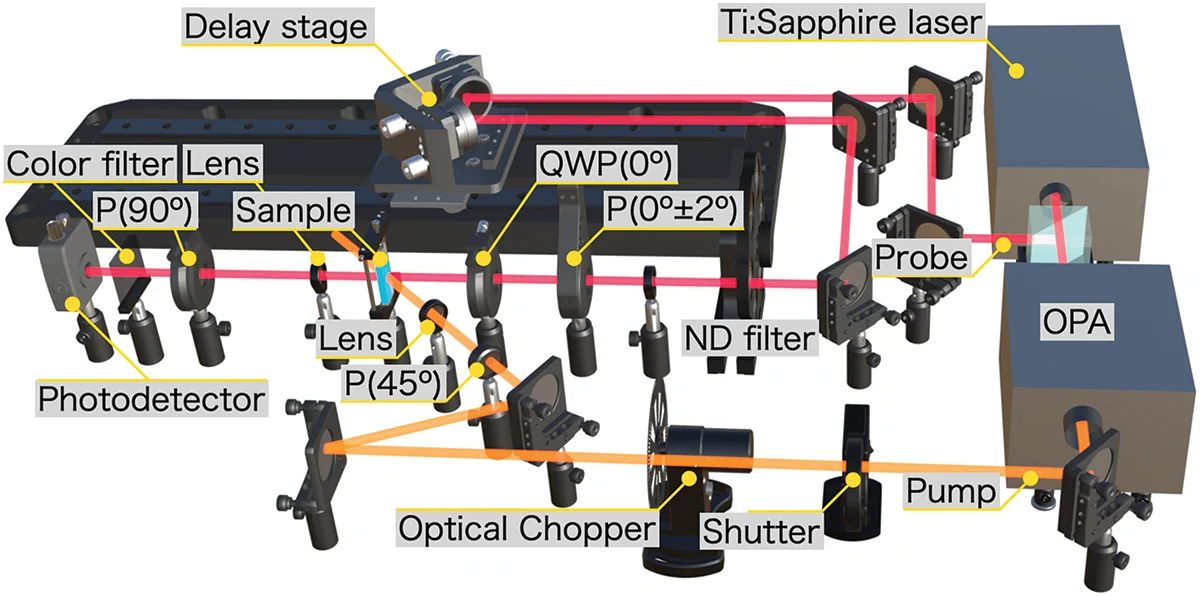
An illustration of experimental setup for OKE spectroscopy. P and QWP refer to polarizer and quarter waveplate, respectively. Source: Thorlabs, Inc. for permission to reproduce CAD-derived renderings of their optomechanical components.

After transmission through the sample, the probe beam passed through an analyzer (P2) oriented at 90° with respect to the initial probe polarization. This crossed-polarizer configuration selectively detects changes in the probe polarization induced by transient birefringence in the sample. A color filter was placed in front of the detector to suppress scattered pump light and eliminate pump-induced artifacts. The transmitted probe intensity was recorded as a function of the pump–probe delay to obtain time-resolved OKE signals.

Temperature-dependent measurements were performed by attaching a thermistor-controlled resistive heater (Thorlabs, USA) to the sample cell to regulate the sample temperature. The cell was wrapped in aluminum foil to improve thermal conduction and ensure a uniform temperature across the cell. Spectra were recorded at 25, 45, 65, and 85°C, with the sample allowed to equilibrate at each temperature before measurement.

### Curve fitting

The reorientational relaxation time τ was obtained by fitting the linear region of each OKE decay to a single exponential,

(1) $$\begin{array}{c} I\left(t\right)=A\exp\left(-\frac{t-t_{0}}{\tau }\right) \end{array}$$

where *I* is the OKE signal intensity, *A* is the amplitude at *t_0_*, *t* is the delay time, *t_0_* is the start of the fitting range, and *τ* is the time constant of the signal decay, taken as the reorientational relaxation time. Because the signal intensity and laser-induced artifacts varied among the datasets, the fitting range was selected separately for each experimental series. The endpoint of each fitting range was defined as the time at which the signal had decayed to approximately ~10^−2^ (a.u.), and had effectively reached the noise floor. The starting point was selected after the fast sub-picosecond response and any laser-induced artifacts had decayed.

For pure water and the PVA solutions, whose decays are themselves sub-picosecond, the fit was started at 0.7 ps. For the temperature-dependent measurements (Figure 4), it was started at 8 ps, because the faster decays at higher temperatures would otherwise leave too few data points if a later start were used. For the concentration-dependent measurements (Figure 5), it was started at 20 ps to exclude a laser-derived noise feature appearing near 20 ps. Regardless of the fitting range selected, each sample yielded consistent decay times, as demonstrated in Figures 4 and 5.

## Results and discussions

### Selection of model amino acids

Optical Kerr effect (OKE) spectroscopy is a third-order nonlinear technique, and the measured signal contains contributions from both solute and solvent. Sensitivity to the reorientational dynamics of the solute therefore requires relatively concentrated solutions, typically on the order of 1 M or higher. We therefore surveyed the aqueous solubilities of a series of amino acids and selected those with the highest solubility (Table S1).

Figure S2 and Table S1 summarize the solubilities of enantiopure (L and D) and racemic forms of representative amino acids in aqueous solution. The solubilities of alanine, valine, and phenylalanine determined here are in good agreement with the classical values reported [15]. The calibration curves obtained from UV–vis spectroscopy are shown in Figure S1. For simple aliphatic amino acids such as alanine and valine (Figure S2 a,b), the solubilities of the L-, D-, and racemic forms are nearly identical within experimental uncertainty.

In contrast, amino acids bearing additional functional groups exhibit more pronounced differences between racemic and enantiopure systems. For arginine, the racemic form shows a slightly higher solubility than the enantiopure forms (Figure S2c), whereas the racemic form is clearly less soluble than the enantiopure forms for phenylalanine and lysine monohydrochloride (Figure S2d,f).

Among the six amino acids, alanine, proline, and lysine monohydrochloride exhibited the highest aqueous solubilities, all dissolving well above 1 M, whereas valine, phenylalanine, and arginine were appreciably less soluble. The former three were therefore selected as model systems: they combine high aqueous solubility with good optical transparency, and span a range of side-chain sizes, allowing the reorientational dynamics to be compared across solutes of different molecular volume. Lysine monohydrochloride, which additionally exhibits a chirality-dependent solubility, further serves as a system in which to examine whether chirality influences the reorientational dynamics. We confirmed the resulting precipitation of racemic lysine monohydrochloride consisted of racemic crystals (Figure S3). The single crystal analysis of obtained D-,L- and racemic crystals are shown in Supporting Information.

### Origin of the picosecond reorientational component in optical Kerr effect spectroscopy

OKE spectroscopy probes the time-dependent anisotropic polarizability of a medium induced by an intense pump pulse. The measured signal consists of two principal contributions: an ultrafast electronic response occurring on sub-picosecond timescales (typically < 1 ps), and a slower nuclear response originating from molecular motions. The latter includes both low-frequency intermolecular vibrational modes and diffusive reorientational relaxation of molecules in solution. While the electronic component reflects the instantaneous electronic polarization, the nuclear component provides direct insight into molecular dynamics and intermolecular interactions in the liquid phase.

Although several groups have reported OKE signals from amino acid derived solutions, most studies have primarily focused on low-frequency vibrational responses in the terahertz or depolarized Raman frequency range [5,16]. In contrast, the reorientational relaxation dynamics of amino acids in solution have received far less attention. In the present work, we focus exclusively on the diffusive reorientational relaxation component, which directly reflects rotational Brownian motion and its coupling to the local hydrodynamic environment.

To verify that the OKE setup is sensitive to solute dynamics under the present experimental conditions, we first measured the OKE signal of pure water (Figure 3(a)). The water signal exhibited a fast relaxation component with a time constant of approximately 0.7 ps, consistent with previously reported ultrafast orientational relaxation of bulk water [8]. This result also confirms that the instrumental response and data analysis procedures are appropriate for resolving sub-picosecond dynamics.

**Figure 3.**
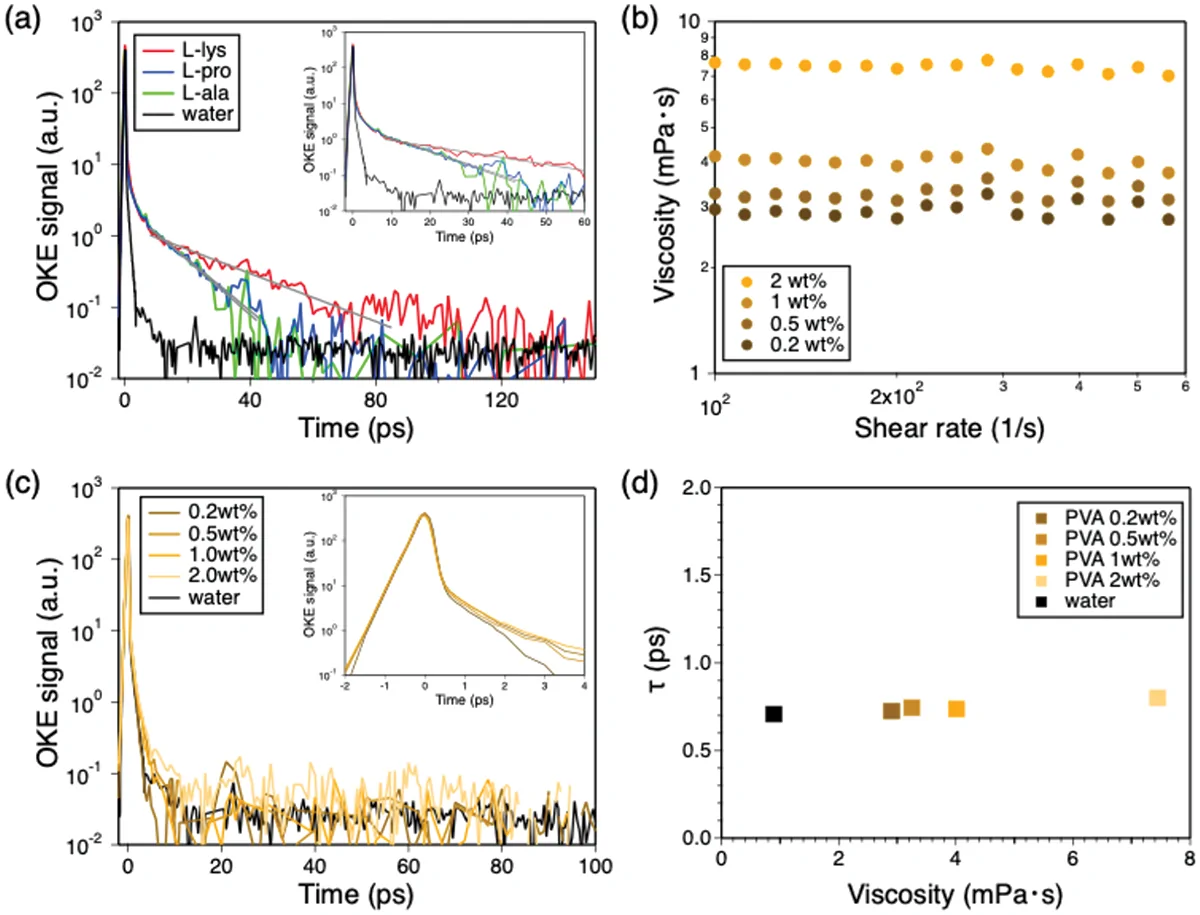
(a) OKE signals of 1 M aqueous L-alanine (green), L-proline (blue), L-lysine monohydrochloride (red), and pure water (black) at 25°C; the inset shows an expanded view over the first 60 ps. (b) Viscosity of aqueous PVA solutions (0.2, 0.5, 1.0, and 2.0 wt%) measured with a rheometer as a function of shear rate. (c) OKE signals of the same PVA solutions and pure water (black); the inset shows the response near time zero. (d) τ plotted against macroscopic viscosity for the PVA solutions, and water. The viscosity of water is taken from the literature values[17].

We then measured aqueous solutions of L-alanine, L-proline, and L-lysine monohydrochloride. In contrast to pure water, each amino acid solution exhibited a clear, slower picosecond component in addition to the fast water response (Figure 3(a)). Because the reorientational relaxation of pure water occurs on a sub-picosecond timescale, the slower component observed in the amino acid solutions initially seems to be attributed to solute reorientation. However, the addition of the solute also increases the solution viscosity and may thereby slow the dynamics of water. It is therefore not immediately clear whether the slow component originates from solute reorientation or from viscosity-slowed water dynamics.

To examine these two possibilities, we used aqueous poly (vinyl alcohol) (PVA; 0.2, 0.5, 1.0, and 2.0 wt%) solutions as control samples. The macroscopic viscosity increased monotonically with PVA concentration, reaching approximately eightfold greater than that for pure water (Figure 3(b)). Since the reorientational relaxation of a polymer of this size (Mw: 85,000 − 124,000) is expected to be far slower than our picosecond observation window, no resolvable reorientational component of the polymer itself should be observed. The OKE response of the PVA solutions is therefore expected to be dominated by the dynamics of water.

The reorientational relaxation time τ of each decay was obtained by Eq. (1). According to the Stokes–Einstein–Debye (SED) relation, this reorientation time is proportional to the hydrodynamic volume of the rotating species and to the friction it experiences,

(2) $$\begin{array}{c} \tau =\frac{\mathrm{V\eta}}{k_{B}T} \end{array}$$

where V, η, $k_{B}$, and T are the effective hydrodynamic molecular volume, the solution viscosity, the Boltzmann constant, and the absolute temperature, respectively [1]. If the slow component in Figure 3(c) arose from water slowed by the increased viscosity, a comparable slow component should also appear in the PVA solutions, particularly at higher PVA concentrations, where the macroscopic viscosity was even greater. However, τ remained almost unchanged from the value of pure water even as the viscosity increased (Figure 3(d), Table S2). Instead, the OKE responses of all PVA solutions remained within 2.5 ps, remaining very close to that of pure water despite the increase in viscosity (Figure 3(c), Table S2). No additional slow picosecond component appeared with increasing PVA concentration.

The slower picosecond component in Figure 3(a) can therefore be assigned primarily to amino acid associated reorientational dynamics rather than to viscosity-slowed bulk water. Accordingly, the extracted relaxation time provides a probe of the local rotational friction associated with the amino acids.

### Reorientation dynamics of amino acids in aqueous solution

The three amino acids were first compared at 1 M and 25°C. Although their macroscopic viscosities are nearly equal (Figure S4), the time constants for reorientational relaxation (*τ*) are 11, 12, and 26 ps for alanine, proline, and lysine monohydrochloride, respectively (Figure 4(a–c)). In particular, lysine monohydrochloride exhibited a relaxation time approximately twice those of alanine and proline. The difference in τ reflects the molecular size as expected from Eq. (2).

**Figure 4.**
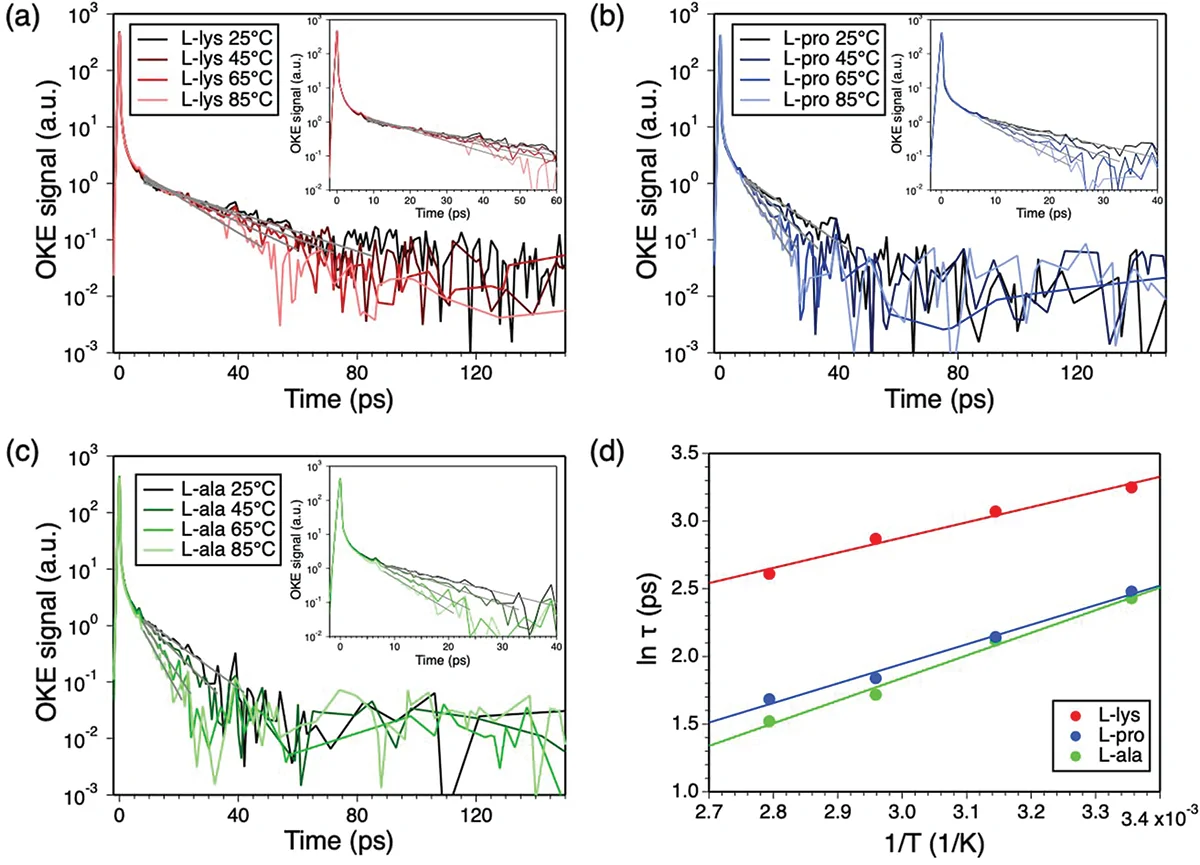
(a–c) OKE signals of 1 M aqueous (a) L-lysine monohydrochloride, (b) L-proline, and (c) L-alanine at 25, 45, 65, and 85°C; the insets show expanded views at early times. (d) τ plotted against η/T, where η is the viscosity of water taken from the literature [17], for L-lysine monohydrochloride (red), L-proline (blue), and L-alanine (green).

For all three amino acids, the decay became faster with increasing temperature (Figure 4(a–c)). According to the SED relation (Eq. (2)), the reorientation time (*τ*) should be linearly related to η/T. The viscosities of the amino-acid solutions were measured using a rotational rheometer (MCR 301, Anton Paar, Austria) and were found to be comparable to that of water at room temperature. Because rheology measurements at high temperature were difficult due to the evaporation and concomitant precipitation, the viscosities at those temperatures were estimated using the correlation reported by Kestin *et al* [17]. When τ was plotted against η/T (Figure 4(d)), proline and alanine showed a nearly linear relationship, whereas lysine monohydrochloride deviated slightly from linearity. This deviation in lysine monohydrochloride solutions may suggest that the local solvation environment of lysine monohydrochloride differs from those of alanine and proline.

According to Eq. (2), the slope of a plot of τ against η/T is proportional to the effective hydrodynamic molecular volume. The obtained slopes for alanine, proline, and lysine monohydrochloride solutions were 3384.7 ± 281, 3277.9 ± 106 and 5711.3 ± 1010 ps K˙mPa^−1^˙s^−1^, respectively. Alanine and proline have comparable molecular sizes, whereas lysine monohydrochloride is substantially larger. To provide a quantitative consistency, we estimated the van der Waals molecular volumes (*V*_*vdw*_) from the optimized DFT geometries using standard atomic van der Waals radii (see the DFT calculation section in the Supporting Information). The obtained volumes were 83.4, 107.0, and 145.5 Å^3^ for alanine, proline, and lysine, respectively. Although these geometric volumes should not be compared directly with the SED-derived effective hydrodynamic volumes (*V*_*eff*_), the relative increase from alanine to lysine is consistent with the larger effective volume obtained from the SED analysis. Furthermore, we estimated the orientational OKE contribution from the molecular polarizability anisotropy. DFT calculations at 700 nm gave much larger polarizability anisotropies for the amino acids than for water (Table S3). Because the orientational OKE contribution is expected to scale approximately with cγ^2^, where c is the molar concentration and γ is the polarizability anisotropy, the amino-acid contribution is estimated to exceed that of water even at 0.25 M [18]. This comparison does not exclude hydration-water or solute–water network contributions, but supports the presence of a substantial amino-acid-associated contribution to the slow picosecond OKE response.

Furthermore, to characterize the temperature dependence of the OKE relaxation from an activation-energy perspective, we performed an Arrhenius-type analysis according to Eq. 3,

(3) $$\begin{array}{c} \begin{array}{c} \tau =\tau_{0}\exp\left(\frac{E_{a}}{\mathrm{RT}}\right) \end{array} \end{array}$$

where $E_{a}$ and R are the apparent activation energy for the observed OKE relaxation and the gas constant, respectively. The results are summarized in Figure S5. The Arrhenius plots were linear for all three amino-acid solutions over the measured temperature range, yielding apparent activation energies of 14.0, 12.4, and 9.5 kJ mol^−1^ for alanine, proline, and lysine monohydrochloride, respectively. The comparable magnitudes of these values indicate that the temperature dependence of the slow OKE relaxation is governed by a similar thermally activated process in the three systems. Notably, lysine monohydrochloride exhibited the longest relaxation time but the smallest apparent activation energy, suggesting that its slower relaxation reflects its larger effective hydrodynamic volume and local rotational friction rather than a larger activation barrier.

We next investigated the concentration dependency using L-lysine monohydrochloride solutions. Figure 5(a) shows the OKE signals of L-lysine monohydrochloride solutions at several concentrations (0.25, 0.50, 1.0, and 2.8 M). As established above, the slow picosecond component reflects the reorientation of lysine monohydrochloride itself rather than that of the surrounding water. The reorientation time τ increased monotonically with concentration (Figure 5(b), Table S5). Within the SED framework, the monotonic increase of τ reflects a growing rotational friction around the solute as the solution becomes more concentrated. The concentration dependence of the viscosity of electrolyte and amino acid solutions is commonly described by the Jones–Dole equation [19–21],

(4) $$\begin{array}{c} \frac{\eta }{\eta_{0}}=1+A\sqrt{c}+\mathrm{Bc} \end{array}$$

**Figure 5.**
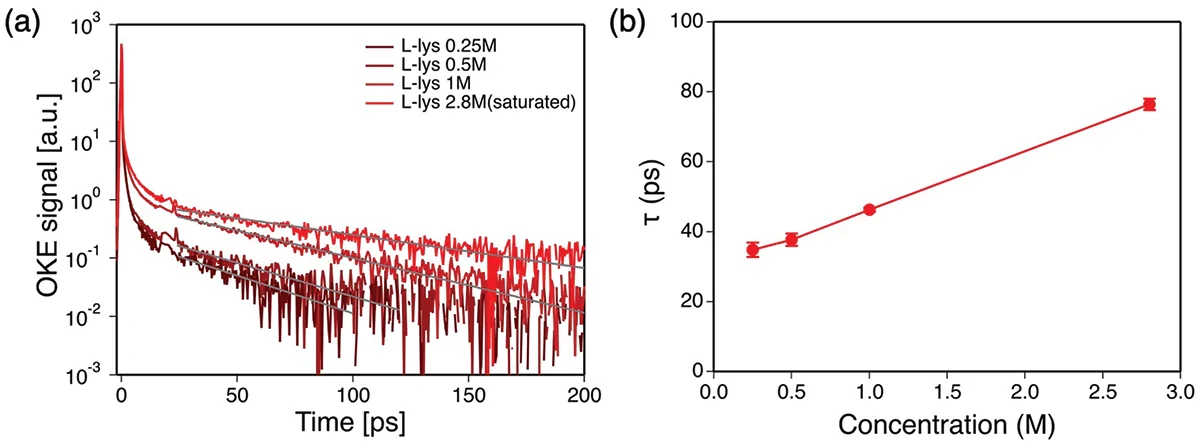
OKE signals at different concentrations for (a) L-lysine monohydrochloride solutions. The concentrations are 0.25, 0.50, 1.0, and 2.8 M, indicated from dark to light colors. (b) τ as a function of concentration.

where η_0_ is the viscosity of the pure solvent and c the solute concentration. The $A\sqrt{c}$ term arises from long-range electrostatic interactions between solutes, whereas the Bc term reflects short-range solute–solvent and solute–solute contacts. Since OKE reports the local friction around the solute, it is the short-range Bc contribution that is most relevant here; the near-linear increase of τ with concentration is consistent with this crowding-dominated, short-range friction.

Additionally, the amplitude A of the slow exponential component (eq.1) extracted from unnormalized OKE traces also increased approximately with lysine monohydrochloride concentration (Table S6). This trend supports a concentration-dependent amino-acid-associated contribution to the slow OKE response, although the amplitude is not used for quantitative decomposition of solute and solvent contributions.

We also examined whether enantiomeric composition affects the concentration-dependent local rotational friction of lysine monohydrochloride. Because the increase in τ with concentration reflects enhanced solute–solute and solute–solvent contacts, racemic and enantiopure solutions could show different relaxation times if homochiral and heterochiral contacts contributed differently to the local friction. However, no measurable difference was observed among D-, L-, and racemic lysine monohydrochloride within experimental uncertainty over the entire concentration range (Figure S6 and Table S5).

## Discussion

The present results indicate that the reorientation time τ obtained from OKE reflects not the macroscopic viscosity of the solution but the local rotational friction in the vicinity of the solute. In the PVA control experiment, increasing the macroscopic viscosity left τ unchanged from the value of pure water. This viscosity increase is therefore likely dominated by polymer-chain-mediated stress relaxation on mesoscopic to macroscopic length scales, while the local rotational environment of water remains nearly unchanged on the sub-picosecond timescale.

In the amino acid solutions, by contrast, τ differed markedly among the solutes even when their macroscopic viscosities were nearly equal, and it increased monotonically with concentration, indicating a concentration-dependent change in the friction experienced at the molecular scale. These observations demonstrate that OKE provides access to local rotational friction, or molecular-scale microviscosity, arising from the solvation environment surrounding the solute and is therefore complementary to conventional rheometry.

The approximately linear concentration dependence of τ is also qualitatively consistent with the Jones–Dole equation, which is associated with short-range solute–solvent and solute–solute interactions, providing additional support for this interpretation.

The present interpretation does not exclude hydration-water or solute–water hydrogen-bond-network dynamics [14]. However, our results are more consistent with the interpretation that the slow component reflects a substantial amino-acid-associated contribution. This assignment is supported by several observations. First, increasing the macroscopic viscosity with PVA did not generate a comparable slow OKE component. Second, the SED-derived *V*_*eff*_ shows the same qualitative size trend as the DFT-estimated *V*_*vdw*_, despite a systematic offset of approximately a factor of two between the static geometric and effective hydrodynamic volumes. Third, DFT calculations show that the polarizability anisotropies of the amino acids are much larger than that of water. Because the heterodyne-detected orientational OKE amplitude is expected to scale approximately with cγ^2^, the amino-acid contribution is estimated to exceed that of water even at 0.25 M. Together with the concentration-dependent increase in the slow-component amplitude, these results support assigning the slow response to amino-acid-associated reorientational relaxation rather than to bulk-water relaxation alone under the present conditions.

We also examined whether enantiomeric composition affects the local rotational friction of lysine monohydrochloride. No measurable difference was observed among D-, L-, and racemic solutions, indicating that any contribution of stereochemically specific contacts to the ensemble-averaged rotational friction is below the sensitivity of the present OKE measurements.

## Conclusion

In this study, we used ultrafast OKE spectroscopy to investigate the reorientational dynamics of three amino acids (alanine, proline, and lysine monohydrochloride) in aqueous solutions, examined in terms of concentration, temperature, and chirality. Using PVA as a control, we showed that the OKE reorientation time τ is unchanged even when the macroscopic viscosity is increased, demonstrating that the picosecond component originates from the rotation of the solute itself. The results indicate that OKE provides information on the local rotational friction associated with the solute and is therefore complementary to rheometry, which measures the macroscopic viscosity of the solution. τ increased in the order of molecular size and rose monotonically with concentration, consistent with the SED law and short-range term of the Jones–Dole equation. These results provide new kinetic insight into the orientational dynamics of amino acids in aqueous solution.

## Supplementary Material

Supplemental Material
