# Supplementary material for: Reorientational dynamics of amino acids in aqueous solution probed by ultrafast optical Kerr effect spectroscopy
Source: Sci Technol Adv Mater. 2026 Sep 21;27(1):2736324. doi: 10.1080/14686996.2026.2736324 (PMC13700517; doi:10.1080/14686996.2026.2736324)
Supplement: Supplementary file 1 [file TSTA_A_2736324_SM8337.docx]

# Supporting Information

Reorientational Dynamics of Amino Acids in Aqueous Solution Probed by Ultrafast Optical Kerr Effect Spectroscopy

*Koichi Murakami^a^, Tomohiro Nobeyama^a,b^, Yuta Koda^a^, Takahiro Sasamori^a^, Kentaro Shiraki^a^, Yohei Yamamoto^a,c^*, and Soh Kushida^a^**

*corresponding author:

Dr. Soh Kushida, Institute of Pure and Applied Sciences, University of Tsukuba, Tsukuba 305-8573, Japan

Prof. Yohei Yamamoto, Institute of Pure and Applied Sciences, Tsukuba Research Center for Energy Materials Science (TREMS) and Hydrogen Boride Research Center, Tsukuba Institute for Advanced Research, University of Tsukuba, Tsukuba 305-8573, Japan

Email: [s-kushida@ims.tsukuba.ac.jp](mailto:s-kushida@ims.tsukuba.ac.jp), yamamoto@ims.tsukuba.ac.jp

**Table of Contents**

**1. General Information	S2**

**2. Experimental Section	S2**

**3. Supporting Figures and Tables	S4**

**4. Single Crystal Analysis	S10**

**5. Calculations for collision frequency	S13**

**6. DFT Calculation	S14**

**7. Supporting Reference	S18**

**1. General Information**

Unless otherwise noted, all chemical compounds and solvents were used as received from either Merck Co., Ltd., WAKENYAKU CO.,LTD. or Tokyo Chemical Industry Co., Ltd. Optical components—including polarizers, wave plates, mirrors, beam splitters, filters, lenses, and others—were purchased from Thorlabs, Inc. Sigma Koki or Newport Corporation. UV–vis absorption spectra were recorded using a commercial spectrophotometer (JASCO V-570). OKE (Optical Kerr Effect) measurements were performed with a home-built apparatus. The delay stage and lock-in amplifier were controlled via Python scripts. Data processing and curve fitting were conducted using Python.

**2*.* Experimental Section**

**Sample Preparation**

**Solutions**: Each amino acid solution was prepared by dissolving the corresponding amino acid in distilled water. The initial amounts of amino acid and water were adjusted to yield a slightly higher concentration than the target value, as dissolution of the amino acid results in a change in the total solution volume and consequently affects the final concentration. The mixture was stirred until complete dissolution. The solution was then adjusted to the final volume using a graduated cylinder to obtain the desired concentration.

**Viscosity measurement**

The viscosities of the aqueous PVA solutions (0.2, 0.5, 1.0, and 2.0 wt%) and of the 1 M amino-acid solutions (L-alanine, L-proline, and L-lysine monohydrochloride) were measured with a rotational rheometer (Anton Paar, MCR 301) at 25 °C. Flow curves were recorded as a function of shear rate, and the viscosity of each solution was taken from the Newtonian region.

**Single Crystal Analysis**:

Single crystals of racemi_a, D, and L were obtained after recrystallization from water solution at room temperature. Intensity data of racemi_a, D, and L were collected on a Bruker APEX-II system using Mo-Ka radiation (λ = 0.71073 Å). The structures were solved using SHELXT-2018^S1^ and refined by a full-matrix least-squares method on *F*^2^ for all reflections using SHELXL-2018.[1] All non-hydrogen atoms were refined anisotropically, and the all hydrogen atoms were refined isotropically. Crystallographic data for racemi_a: C_6_H_15_ClN_2_O_2_, FW 182.65, crystal size 0.25×0.15×0.10 mm^3^, temperature –170 ˚C, monoclinic, space group *P*2_1_/a (#14), *a* = 8.5308(1) Å, *b* = 11.1431(1) Å, *c* = 9.1365(1) Å, *β* = 105.8894(14)˚, *V* = 835.327(16) Å^3^, *Z* = 4, *D*_calcd_ = 1.452 g cm^–3^, *μ* = 0.412 mm^–1^, *θ*_max_ = 33.629˚, 33741 reflections measured, 3206 independent reflections (*R*_int_ = 0.0148), 160 parameters refined, GOF = 1.115, completeness = 99.3%, *R*_1_ [*I* > 2*σ*(*I*)] = 0.0201, w*R*_2_ (all data) = 0.0560, largest diff. peak and hole 0.510 and –0.227 e Å^–3^. D: C_6_H_19_ClN_2_O_4_, FW 218.68, crystal size 0.20×0.15×0.12 mm^3^, temperature –170 ˚C, monoclinic, space group *P*2_1_ (#4), *a* = 5.8646(1) Å, *b* = 13.2180(2) Å, *c* = 7.4120(1) Å, *β* = 97.9459(12)˚, *V* = 569.049(15) Å^3^, *Z* = 2, *D*_calcd_ = 1.276 g cm^–3^, *μ* = 0.326 mm^–1^, *θ*_max_ = 33.609˚, 15909 reflections measured, 4084 independent reflections (*R*_int_ = 0.0106), 194 parameters refined, GOF = 1.089, completeness = 99.5%, *R*_1_ [*I* > 2*σ*(*I*)] = 0.0166, w*R*_2_ (all data) = 0.0448, largest diff. peak and hole 0.275 and –0.190 e Å^–3^. L: C_6_H_19_ClN_2_O_4_, FW 218.68, crystal size 0.20×0.18×0.05 mm^3^, temperature –170 ˚C, monoclinic, space group *P*2_1_ (#4), *a* = 5.8691(1) Å, *b* = 13.2195(3) Å, *c* = 7.4146(2) Å, *β* = 98.010(2)˚, *V* = 569.66(2) Å^3^, *Z* = 2, *D*_calcd_ = 1.275 g cm^–3^, *μ* = 0.326 mm^–1^, *θ*_max_ = 33.540˚, 19051 reflections measured, 4034 independent reflections (*R*_int_ = 0.0122), 194 parameters refined, GOF = 1.133, completeness = 100.0%, *R*_1_ [*I* > 2*σ*(*I*)] = 0.0176, w*R*_2_ (all data) = 0.0475, largest diff. peak and hole 0.233 and –0.267 e Å^–3^.

**3. Supporting Figures and Tables**

**Figure S1.** Calibration curves of (a) alanine (207 nm), (b) valine (207 nm), (c) arginine (221 nm), (d) phenylalanine (257 nm), (e) proline (213 nm), and (f) lysine (217 nm) obtained by UV–Vis spectroscopy. The data were fitted using least-squares linear regression.

**Figure S2.** Solubilities of enantiopure (L and D) and racemic amino acids in aqueous solution. The amino acids are (a) alanine, (b) valine, (c) arginine, (d) phenylalanine, and (e) proline, (f) lysine monohydrochloride. The molecular structures of each amino acid are shown above the corresponding plots. Error bars represent standard deviations obtained from repeated measurements.

**Figure S3.** Photographs of aqueous lysine monohydrochloride solutions prepared at an initial concentration of 2.8 M: (a) enantiopure (L-) and (b) racemic.

**Figure S4.** Viscosity of 1 M aqueous L-alanine (green), L-proline (blue), and L-lysine monohydrochloride (red) at 25 °C, measured by rheometer as a function of shear rate. The three solutions have nearly identical viscosities.

**Figure S5.** Arrhenius plots of the reorientation time for L-lysine monohydrochloride (red), L-proline (blue), and L-alanine (green); the solid lines are linear fits, yielding apparent activation energies of 14.0, 12.4, and 9.5 kJ mol-1 for alanine, proline, and lysine monohydrochloride, respectively.

**Figure S6.** OKE signals at different concentrations for (a) D-, (b) L-, and (c) Rac-lysine monohydrochloride solutions. The concentrations are 0.25, 0.50, 1.0, and 2.8 M, indicated from dark to light colors. (d) τ as a function of concentration.

**Table S1.** Solubilities of enantiopure (L and D) and racemic amino acids in aqueous solution.

| Amino acids | S_L_ (M) | S_D_ (M) | S_Rac_ (M) |
| --- | --- | --- | --- |
| Alanine | 1.691 $\pm$ 0.009 | 1.640 $\pm$ 0.009 | 1.680 $\pm$ 0.009 |
| Valine | 0.560 $\pm$ 0.009 | 0.553 $\pm$ 0.009 | 0.567 $\pm$ 0.009 |
| Arginine | 0.907 $\pm$ 0.007 | 1.062 $\pm$ 0.016 | 1.085 $\pm$ 0.020 |
| Phenylalanine | 0.163 $\pm$ 0.0005 | 0.164 $\pm$ 0.0005 | 0.0772 $\pm$ 0.0004 |
| Proline | 6.046 ± 0.010 | 6.278 ± 0.001 | 6.089 ± 0.010 |
| Lysine・HCl | 2.756 $\pm$ 0.005 | 2.759 $\pm$ 0.005 | 1.198 $\pm$ 0.001 |

**Table S2.** Reorientational relaxation time of pure water and aqueous PVA solutions.

| Sample | τ (ps) |
| --- | --- |
| Water | 0.7 $\pm$ 0.01 |
| PVA 2wt% | 0.8 $\pm$ 0.01 |
| PVA 1wt% | 0.7 $\pm$ 0.01 |
| PVA 0.5wt% | 0.7 $\pm$ 0.01 |
| PVA 0.2wt% | 0.7 $\pm$ 0.01 |

**Table S3.** DFT-derived polarizability parameters at 700 nm. The polarizability anisotropy γ was obtained from the frequency-dependent polarizability tensor α(−ω;ω) at 700 nm.

| Sample | γ / au | γ² / au² |
| --- | --- | --- |
| L-lysine | 23.63 | 558.52 |
| L-alanine | 18.8 | 353.45 |
| L-proline | 20.35 | 414 |
| water | 0.335 | 0.112 |

**Table S4.** Reorientational relaxation time of 1 M aqueous L-alanine, L-proline, and L-lysine monohydrochloride at 25, 45, 65, and 85 °C.

| Sample | 25 ℃ (ps) | 45 ℃ (ps) | 65 ℃ (ps) | 85 ℃ (ps) |
| --- | --- | --- | --- | --- |
| L-lysine | 25.8 $\pm$ 0.8 | 21.6 $\pm$ 0.6 | 17.5 $\pm$ 0.4 | 13.5 $\pm$ 0.4 |
| L-alanine | 11.3 $\pm$ 0.5 | 8.2 $\pm$ 0.3 | 5.4 $\pm$ 0.2 | 4.6 $\pm$ 0.3 |
| L-proline | 11.8 $\pm$ 0.4 | 8.4 $\pm$ 0.4 | 6.2 $\pm$ 0.2 | 5.2 $\pm$ 0.2 |

**Table S5.** Reorientational relaxation time of lysine monohydrochloride aqueous solutions with different enantiomeric ratios and concentrations.

| Sample | 0.25 M (ps) | 0.5 M (ps) | 1 M (ps) | 2.8 M (ps) |
| --- | --- | --- | --- | --- |
| L-lysine | 33.6 $\pm$ 2.0 | 37.7 $\pm$ 1.8 | 46.3 $\pm$ 0.6 | 76.4 $\pm$ 1.6 |
| D-lysine | 37.2 $\pm$ 3.9 | 37.7 $\pm$ 1.5 | 46.9 $\pm$ 0.8 | 77.1 $\pm$ 1.5 |
| Rac-Lysine | 39.8 $\pm$ 3.1 | 37.1 $\pm$ 1.1 | 45.2 $\pm$ 1.2 | 79.6 $\pm$ 2.65 |

**Table S6.** Slow-component amplitudes A obtained from single-exponential fits to the unnormalized OKE traces of D-, L-, and racemic lysine monohydrochloride solutions at different concentrations.

| Sample | 0.25 M (a.u.) | 0.5M (a.u.) | 1M (a.u.) | 2.8M (a.u.) |
| --- | --- | --- | --- | --- |
| L-lysine | 0.150 ± 0.008 | 0.198 ± 0.006 | 0.591 ± 0.007 | 0.730 ± 0.011 |
| D-lysine | 0.066 ± 0.004 | 0.259 ± 0.008 | 0.402 ± 0.006 | 0.727 ± 0.011 |
| Rac-Lysine | 0.108 ± 0.005 | 0.271 ± 0.005 | 0.645 ± 0.012 | 0.714 ± 0.016 |

**Table S7.** Effective hydrodynamic volumes derived from the SED analysis and DFT-estimated van der Waals volumes of alanine, proline, and lysine monohydrochloride.

| Sample | Slope S  (ps·K·mPa^-1^·s^-1^) | V_eff_  (Å^3^)^a^ | V_vdw_  (Å^3^)^b^ |
| --- | --- | --- | --- |
| L-alanine | 3384.7 ± 281 | 46.7 ± 3.9 | 83.4 |
| L-proline | 3277.9 ± 106 | 45.3 ± 1.5 | 107 |
| L-lysine monohydrochloride | 5711.3 ± 1010 | 78.9 ± 13.9 | 145.5 |

a: Estimated from OKE experiments. b: Estimated from DFT calculations.

**4. Single Crystal Analysis**

**Table S8**. Crystal structure, crystal data and structure refinement for L-lysine.

Identification code	L

Empirical formula	C6 H19 Cl N2 O4

Formula weight	218.68

Temperature	103(2) K

Wavelength	0.71073 Å

Crystal system	Monoclinic

Space group	P2**_1_ (#4)**

Unit cell dimensions	a = 5.86910(10) Å	a= 90°.

b = 13.2195(3) Å	b= 98.010(2)°.

c = 7.4146(2) Å	g = 90°.

Volume	569.66(2) Å^3^

Z	2

Density (calculated)	1.275 Mg/m^3^

Absorption coefficient	0.326 mm^-1^

F(000)	236

Crystal size	0.200 x 0.180 x 0.050 mm^3^

Theta range for data collection	2.774 to 33.540°.

Index ranges	-9<=h<=9, -20<=k<=20, -11<=l<=11

Reflections collected	19051

Independent reflections	4034 [R(int) = 0.0122]

Completeness to theta = 25.242°	100.0 %

Refinement method	Full-matrix least-squares on F^2^

Data / restraints / parameters	4034 / 1 / 194

Goodness-of-fit on F^2^	1.133

Final R indices [I>2sigma(I)]	R1 = 0.0176, wR2 = 0.0473

R indices (all data)	R1 = 0.0180, wR2 = 0.0475

Absolute structure parameter	0.004(8)

Extinction coefficient	n/a

Largest diff. peak and hole	0.233 and -0.267 e.Å^-3^

**Table S9**. Crystal structure, crystal data and structure refinement for D-lysine.

Identification code	D

Empirical formula	C6 H19 Cl N2 O4

Formula weight	218.68

Temperature	103(2) K

Wavelength	0.71073 Å

Crystal system	Monoclinic

Space group	*P*2**_1_ (#4)**

Unit cell dimensions	a = 5.86460(10) Å	a= 90°.

b = 13.2180(2) Å	b= 97.9459(12)°.

c = 7.41200(10) Å	g = 90°.

Volume	569.049(15) Å^3^

Z	2

Density (calculated)	1.276 Mg/m^3^

Absorption coefficient	0.326 mm^-1^

F(000)	236

Crystal size	0.200 x 0.150 x 0.120 mm^3^

Theta range for data collection	2.775 to 33.609°.

Index ranges	-9<=h<=9, -20<=k<=19, -11<=l<=10

Reflections collected	15909

Independent reflections	4084 [R(int) = 0.0106]

Completeness to theta = 25.242°	99.5 %

Refinement method	Full-matrix least-squares on F^2^

Data / restraints / parameters	4084 / 1 / 194

Goodness-of-fit on F^2^	1.089

Final R indices [I>2sigma(I)]	R1 = 0.0166, wR2 = 0.0448

R indices (all data)	R1 = 0.0167, wR2 = 0.0448

Absolute structure parameter	0.008(8)

Extinction coefficient	n/a

Largest diff. peak and hole	0.275 and -0.190 e.Å^-3^

**Table S10**. Crystal structure, crystal data and structure refinement for Rac-lysine

Identification code	racemi_a

Empirical formula	C6 H15 Cl N2 O2

Formula weight	182.65

Temperature	103(2) K

Wavelength	0.71073 Å

Crystal system	monoclinic

Space group	*P*2_1_/a (#14)

Unit cell dimensions	a = 8.53080(10) Å	a= 90°.

b = 11.14310(10) Å	b= 105.8894(14)°.

c = 9.13650(10) Å	g = 90°.

Volume	835.327(16) Å^3^

Z	4

Density (calculated)	1.452 Mg/m^3^

Absorption coefficient	0.412 mm^-1^

F(000)	392

Crystal size	0.250 x 0.150 x 0.100 mm^3^

Theta range for data collection	2.952 to 33.629°.

Index ranges	-13<=h<=13, -17<=k<=17, -14<=l<=14

Reflections collected	33741

Independent reflections	3206 [R(int) = 0.0148]

Completeness to theta = 25.242°	99.3 %

Refinement method	Full-matrix least-squares on F^2^

Data / restraints / parameters	3206 / 0 / 160

Goodness-of-fit on F^2^	1.115

Final R indices [I>2sigma(I)]	R1 = 0.0201, wR2 = 0.0557

R indices (all data)	R1 = 0.0205, wR2 = 0.0560

Extinction coefficient	n/a

Largest diff. peak and hole	0.510 and -0.227 e.Å^-3^

**5. Calculations for collision frequency**

To estimate the frequency of intermolecular encounters in concentrated lysine solutions, we employed a diffusion-controlled collision model based on the Stokes–Einstein relation. The translational diffusion coefficient $D$ was approximated using

$$D=\frac{k_{B}T}{6\pi\eta r_{h}}$$

where $k_{B}$ is the Boltzmann constant, $T$ is the absolute temperature (298 K), $\eta$ is the solution viscosity, and $r_{h}$ is the hydrodynamic radius of lysine.

For the 2.8 M lysine solution, we assumed $\eta=10$ mPa·s and $r_{h}=0.5$nm,[2] yielding $D\approx4.4\times{10}^{-11}$m² s⁻¹. The diffusion-controlled collision rate constant for identical molecules was estimated using the Smoluchowski expression:

$$k_{\text{diff}}=8\pi DR$$

where $R$ is the encounter distance, approximated as $R=2r_{h}$. Under these assumptions, $k_{\text{diff}}\approx1.1\times{10}^{-18}$m³ s⁻¹. The collision frequency per molecule at concentration ($C$) was then estimated as

$$f\approx k_{\text{diff}} n$$

where $n=1000N_{A}C$ is the number density with $N_{A}$ denoting Avogadro’s constant (6.022 × 10²³ mol⁻¹). For $C=2.8$ mol L⁻¹, this yields $f\sim2\times{10}^{9}$ s⁻¹, corresponding to an average intermolecular encounter every ~ 0.5 ns. This estimated collision timescale is many orders of magnitude shorter than the characteristic timescale of crystallization (typically ~hours), indicating that nucleation cannot be limited by simple diffusive encounters but instead requires rare, orientation-specific configurations.

**6. DFT calculation**

All ab initio and density functional theory (DFT) calculations were performed by using Gaussian 16 Rev.A.03 or Rev.B.01 and results were visualized with Gaussview6^3, 4^. All geometries were optimized using density functional theory (DFT) at the B3LYP/aug-cc-pvtz level of theory without any symmetry assumptions. Harmonic vibration frequency analysis was performed at the same level of theory to verify all stationary points as local minima (with no imaginary frequency). The size of molecule was calculated from the optimized coordination by getting diameter value as the longest distance between the center of gravity and atoms for each molecule. As for the polarity, we conducted a single point calculation with the following keywords: Polar, pop=full, CPHF=Rdfreq, int=grid=ultrafine.

**L-alanine**

Optimized Cartesian coordinates (Å)

N	1.23818300	-1.15346700	0.12910900

C	0.58092100	0.17916900	0.40804000

C	1.25996300	1.30480000	-0.35384100

C	-0.92409800	-0.01041100	0.03744900

O	-1.22438300	-1.15702700	-0.39783500

O	-1.66377700	0.97019700	0.21551900

H	0.42124700	-1.72200000	-0.20543900

H	1.94823900	-1.10479900	-0.59791500

H	1.65555300	-1.57775600	0.95292000

H	0.64763200	0.35078700	1.47948300

H	0.74057000	2.23627400	-0.14418500

H	2.30056900	1.41523900	-0.04986900

H	1.22347600	1.12980800	-1.43011500

Frequency-dependent polarizability tensor at 700 nm

Alpha(-ω;ω), ω = 700 nm

Dipole polarizability tensor in the Gaussian molecular coordinate system.

(au)	(10**-24 esu)	(10**-40 SI)

iso	0.636504D+02	0.943202D+01	0.104945D+02

aniso	0.188002D+02	0.278591D+01	0.309974D+01

xx	0.514999D+02	0.763149D+01	0.849118D+01

yx	0.134635D+01	0.199509D+00	0.221984D+00

yy	0.688172D+02	0.101977D+02	0.113464D+02

zx	0.147947D+01	0.219234D+00	0.243931D+00

zy	-0.150418D+01	-0.222896D+00	-0.248006D+00

zz	0.706342D+02	0.104669D+02	0.116460D+02

**L-proline**

Optimized Cartesian coordinates (Å)

N	0.70763700	-1.02510600	0.57897900

C	1.98636600	-0.60838300	-0.10497600

C	1.61771400	0.70293800	-0.78753900

C	0.65546200	1.36087400	0.20940100

C	-0.18091400	0.19405500	0.75172300

C	-1.48557400	-0.09143900	-0.05161400

O	-2.35288600	0.79737100	-0.01719800

O	-1.51760300	-1.20237800	-0.64833000

H	0.88318500	-1.51508600	1.45118700

H	0.10628300	-1.62382100	-0.02917000

H	2.73738700	-0.46091000	0.66643800

H	2.30674100	-1.40165300	-0.77305700

H	1.11908900	0.50969900	-1.73774300

H	2.49938900	1.30744900	-0.98609900

H	0.01801400	2.11519400	-0.24327400

H	1.21415900	1.83021400	1.01916100

H	-0.43211800	0.30643900	1.80195900

Frequency-dependent polarizability tensor at 700 nm

Alpha(-ω;ω), ω = 700 nm

Dipole polarizability tensor in the Gaussian molecular coordinate system.

(au)	(10**-24 esu)	(10**-40 SI)

iso	0.843808D+02	0.125039D+02	0.139125D+02

aniso	0.203471D+02	0.301513D+01	0.335478D+01

xx	0.739220D+02	0.109541D+02	0.121881D+02

yx	-0.258368D+01	-0.382862D+00	-0.425991D+00

yy	0.857067D+02	0.127004D+02	0.141311D+02

zx	0.542775D+01	0.804310D+00	0.894916D+00

zy	-0.214191D+01	-0.317398D+00	-0.353153D+00

zz	0.935138D+02	0.138573D+02	0.154183D+02

**L-lysine**

Optimized Cartesian coordinates (Å)

N	1.72186400	1.78677300	-0.03799100

C	1.50327100	0.49090900	0.71126900

C	0.02819800	0.10090000	0.78179100

C	-0.62934600	-0.20728700	-0.56804400

C	-2.08322100	-0.68140700	-0.45408000

C	-3.07246600	0.39868300	-0.02529000

N	-4.44337600	-0.13493300	0.00161300

C	2.41846200	-0.56075400	0.00675100

O	3.08215900	-0.11931600	-0.97214500

O	2.41015100	-1.70586300	0.48552300

H	2.08829200	2.53149100	0.54841700

H	2.43180300	1.49818600	-0.75444800

H	0.88459100	2.13089800	-0.50168500

H	1.88550900	0.64236400	1.71776000

H	-0.02256700	-0.78520700	1.41480900

H	-0.51360500	0.88914100	1.30770400

H	-0.04972500	-0.98353700	-1.07142600

H	-0.60130100	0.66822200	-1.22542100

H	-2.40482000	-1.07244300	-1.42180000

H	-2.13453200	-1.51785000	0.25077000

H	-3.04818400	1.21626400	-0.74966800

H	-2.77252900	0.82154800	0.94044200

H	-5.09669300	0.59711100	0.25509000

H	-4.52352100	-0.84390700	0.72270100

Frequency-dependent polarizability tensor at 700 nm

Alpha(-ω;ω), ω = 700 nm

Dipole polarizability tensor in the Gaussian molecular coordinate system.

(au)	(10**-24 esu)	(10**-40 SI)

iso	0.116349D+03	0.172411D+02	0.191833D+02

aniso	0.236330D+02	0.350205D+01	0.389656D+01

xx	0.120859D+03	0.179095D+02	0.199270D+02

yx	0.104114D+02	0.154281D+01	0.171660D+01

yy	0.111433D+03	0.165127D+02	0.183729D+02

zx	-0.744537D+01	-0.110329D+01	-0.122758D+01

zy	0.836782D-01	0.123998D-01	0.137967D-01

zz	0.116753D+03	0.173011D+02	0.192500D+02

**Water**

Optimized Cartesian coordinates (Å)

H	0.00000000	-0.77781100	1.52378900

O	0.00000000	-0.02950700	0.91724400

H	0.00000000	0.74613100	1.48881800

Frequency-dependent polarizability tensor at 700 nm

Alpha(-ω;ω), ω = 700 nm

Dipole polarizability tensor in the Gaussian molecular coordinate system.
                  (au)         (10**-24 esu)      (10**-40 SI)
   iso        0.105481D+02      0.156306D+01      0.173914D+01
   aniso      0.335288D+00      0.496845D-01      0.552815D-01
   xx         0.104277D+02      0.154523D+01      0.171930D+01
   yx         0.000000D+00      0.000000D+00      0.000000D+00
   yy         0.107714D+02      0.159615D+01      0.177596D+01
   zx         0.000000D+00      0.000000D+00      0.000000D+00
   zy         0.129327D-02      0.191642D-03      0.213231D-03
   zz         0.104451D+02      0.154781D+01      0.172217D+01

The polarizability anisotropy was calculated from the frequency-dependent polarizability tensor at 700 nm as

$$\gamma^{2}=\frac{1}{2}[\left( \alpha_{xx}-\alpha_{yy} \right)^{\boldsymbol{2}}+\mathbf{(}\left( \alpha_{yy}-\alpha_{zz} \right)^{\boldsymbol{2}}+\left( \alpha_{zz}-\alpha_{xx} \right)^{\boldsymbol{2}}]+3\left( {\alpha_{xy}}^{2}+{\alpha_{xz}}^{2}+{\alpha_{yz}}^{2} \right)$$

The orientational OKE contribution was then estimated using the approximate scaling$A_{\mathrm{OKE}}\propto{c\gamma}^{2}$, where c is the molar concentration.

**7. References**

[1]	Usón I, Sheldrick GM. An introduction to experimental phasing of macromolecules illustrated by *SHELX* ; new autotracing features. Acta Crystallogr D Struct Biol. 2018;74(2):106–116.

[2]	Patil, KC, Gowardipe A. Volumetric, ultrasonic and viscometric studies on L-lysine mono-hydrochloride in aqueous medium at different temperatures. IJRAT. 2019;7(4):453–458.

[3]	M. J. Frisch, G. W. T., H. B. Schlegel, G. E. Scuseria, ; M. A. Robb, J. R. C., G. Scalmani, V. Barone, ; G. A. Petersson, H. N., X. Li, M. Caricato, A. V. Marenich, ; J. Bloino, B. G. J., R. Gomperts, B. Mennucci, H. P. Hratchian, ; J. V. Ortiz, A. F. I., J. L. Sonnenberg, D. Williams-Young, ; F. Ding, F. L., F. Egidi, J. Goings, B. Peng, A. Petrone, ; T. Henderson, D. R., V. G. Zakrzewski, J. Gao, N. Rega, ; G. Zheng, W. L., M. Hada, M. Ehara, K. Toyota, R. Fukuda, ; J. Hasegawa, M. I., T. Nakajima, Y. Honda, O. Kitao, H. Nakai, ; T. Vreven, K. T., J. A. Montgomery, Jr., J. E. Peralta, ; et al. conputer code GAUSSIAN 16 revision A.03, Gaussian, Inc 2016.

[4]	M. J. Frisch, G. W. T., H. B. Schlegel, G. E. Scuseria, ; M. A. Robb, J. R. C., G. Scalmani, V. Barone, ; G. A. Petersson, H. N., X. Li, M. Caricato, A. V. Marenich, ; J. Bloino, B. G. J., R. Gomperts, B. Mennucci, H. P. Hratchian, ; J. V. Ortiz, A. F. I., J. L. Sonnenberg, D. Williams-Young, ; F. Ding, F. L., F. Egidi, J. Goings, B. Peng, A. Petrone, ; T. Henderson, D. R., V. G. Zakrzewski, J. Gao, N. Rega, ; G. Zheng, W. L., M. Hada, M. Ehara, K. Toyota, R. Fukuda, ; J. Hasegawa, M. I., T. Nakajima, Y. Honda, O. Kitao, H. Nakai, ; T. Vreven, K. T., J. A. Montgomery, Jr., J. E. Peralta, ; et al. conputer code GAUSSIAN 16 revision B.01, Gaussian, Inc 2016
